# DIZZYNET 2020: basic and clinical vestibular research united

**DOI:** 10.1007/s00415-020-10317-4

**Published:** 2020-11-18

**Authors:** Andreas Zwergal, Raymond van de Berg, Marianne Dieterich

**Affiliations:** 1 Department of Neurology, Ludwig-Maximilians-University, Marchioninistrasse 15, 81377 Munich, Germany; 2 German Center for Vertigo and Balance Disorders, DSGZ, Ludwig-Maximilians-University, Munich, Germany; 3 Department of ENT, Maastricht University Medical Center, Maastricht, The Netherlands

**Keywords:** European dizzynet, Translational vestibular research, Vertigo, Dizziness, Balance disorders

Our current clinical knowledge of the vestibular system has greatly benefitted from more than two centuries of basic experimental research on the anatomical structure of peripheral and central vestibular networks, vestibular signaling properties and their functional implications for gaze, posture, locomotion, and spatial orientation. Clinical standard applications, such as testing of the vestibular-ocular reflex by head impulses or quantification of otolith function by vestibular evoked myogenic potentials, are based on evidence gained from experiments in a variety of vertebrate species. The almost identical structure of sensory endorgans and neuronal pathways in different vertebrates is the prerequisite for this translation [1]. The vestibular research community has the particular chance to take advantage of this fact and use the scope of novel methods and techniques to foster interdisciplinary research from bench to bedside and back. The DIZZYNET aims to provide a platform for exchange between basic, translational and clinical researchers, who specialize in vestibular, balance, and gait disorders [2]. At the sixth DIZZYNET meeting, which took place in Sonnenhausen near Munich in October 2019, experts from 20 European countries and the USA discussed innovations in different fields of vestibular research. We have collected selected contributions presented at this meeting in the current DIZZYNET issue of the Journal of Neurology:

In a basic research experiment, Soupiadou and colleagues report instantaneous effects of unilateral XIIIth nerve transection on the vestibulo-ocular and optokinetic reflexes in Xenopus laevis tadpoles, which suggest a combined plasticity of visuo-vestibular neuronal networks. Two translational research articles describe the effects of static magnetic field stimulation on cerebral resting-state networks and passive whole-body accelerations on EEG microstates. Another EEG-based experiment by McAssey and team indicates alpha band changes during ongoing visually induced self-motion perception with a topographical difference in left- and right-handers.

Several clinical research articles focus on diagnostic work-up and management of acute vestibular and ocular motor disorders. Machner and colleagues propose a clinical risk stratification for the proper use of neuroimaging in dizzy patients in the emergency room. While patients with HINTS-negative acute vestibular syndrome almost never have stroke, about 50% of patients with acute imbalance and cardiovascular risk factors (ABCD^2^ ≥ 4) suffer from stroke. Ahmadi et al. show the feasibility of modern machine-learning methods to separate acute central from acute peripheral vestibular disorders with a high diagnostic accuracy. In another paper, Kremmyda and colleagues report a three-feature rule, which can differentiate peripheral from central causes of acute binocular diplopia with a positive predictive value of 100%: accompanying vertigo/dizziness, central ocular motor signs, and pathological monocular deviation of the subjective visual vertical on the non-paretic eye. Episodic vestibular disorders, including Menière’s disease and vestibular migraine, are the topic of several other articles. The methodology and benefit of hydrops imaging are discussed in two papers (Gerb et al. van der Lubbe et al.). Martin, de Joode and colleagues report first data from the practical application of a novel app-based diary for vestibular disorders (named DIZZYQuest). They show that the digital recording of frequency, duration and symptom characteristics of vestibular attacks is feasible and promising for future studies. In the field of chronic vestibular disorders, research on bilateral vestibulopathy (BVP) gains the most interest. The chief physical, cognitive, and emotional complaints are described from the patient perspective in a paper by Lucieer et al. The complex symptomatology of BVP is further illustrated by two contributions from the group of Christophe Lopez, which report subtle alterations of interoception and emotional body-maps in BVP. Boutabla et al. show evidence for the simultaneous stimulation of vestibulo-spinal, -ocular and -perceptive pathways in three BVP patients with vestibular implants.

The above-mentioned articles demonstrate the broad spectrum of topics from basic research to clinical application and from diagnostics to therapy represented by the DIZZYNET members. This supplement also includes data from multinational surveys among European ENT specialists (Weckel et al.) and vestibular therapists (Meldrum et al.), which document the clinical view of current and future research practice from an interdisciplinary perspective.
